# The Consequences of the Pandemic for Subjective Well-Being: Data for Improving Policymaking

**DOI:** 10.3390/ijerph192416572

**Published:** 2022-12-09

**Authors:** Lina Martínez, Eduardo Lora, Andres David Espada

**Affiliations:** 1 Business School, Universidad Icesi & POLIS, Cali 760031, Colombia; 2 Center for International Development, Harvard University, Cambridge, MA 02138, USA; 3 Department of Economics, Universidad Icesi, Cali 760031, Colombia; 4 International Center for Tropical Agriculture (CIAT), Palmira 763537, Colombia

**Keywords:** pandemic, subjective well-being, gender, health

## Abstract

The pandemic has affected people’s lives and emotions in profound ways, which governments ignore at their peril. Among the often disregarded consequences of the pandemic, especially in developing countries, are its toll on subjective well-being and its implications for health policymaking. This paper uses a battery of surveys with over 1800 observations collected in 2019 and 2020, which inform on many aspects of subjective well-being before and during the pandemic in Cali, Colombia. The results show a dramatic and widespread reduction in life satisfaction in several dimensions of well-being beyond health, and not just among those directly affected by COVID-19. This analysis focuses on differences in well-being by gender and health status, providing information about gender variances and differences in subjective well-being between those who experienced and those who did not experience physical illness (including the COVID-19 infection) during the pandemic. This analysis aims at contributing to the body of research that studies the consequences of the pandemic for life satisfaction and well-being, in the context of a city experiencing profound social unrest during the pandemic.

## 1. Introduction

The pandemic caused by COVID-19 is possibly the most important shock recent generations have lived through. The aftermath and the long-term consequences are still in the making, and it is difficult to predict its many impacts and ramifications. So far, we have witnessed a rising number of deaths, a severe economic contraction, and disparities among governments in dealing with the crisis [1]. An additional underappreciated consequence of the pandemic is the toll on happiness and subjective well-being. In Europe, when put in monetary terms, the negative impacts on well-being during the pandemic were 3,5 times the losses in GDP [2]. Any sustainable policy intervention for recovery after the pandemic not only depends on saving lives and recovering the economy. Conditions affecting mental health, such as anxiety, stress, and low morale, have become so common that they may affect aggregate productivity. The population’s subjective well-being is often overlooked by policymakers, despite all the potentially devastating implications [3].

Data on subjective well-being and life satisfaction are relatively new in the policy domain, explaining why subjective well-being indicators are not prioritized in many governments’ agendas. Since 2012, OECD countries have adopted standardized metrics to measure different aspects of subjective well-being, including evaluative measures (life satisfaction), affect measures (related to the experience of positive and negative emotions), and eudemonic measures (related to people’s psychological functioning). National statistical offices in developed countries are increasingly interested in adding to and using the information provided by subjective well-being metrics [4,5]. Nevertheless, governments in developing countries are only just arriving at this discussion, and they are poorly equipped to make use of the power of subjective well-being metrics to understand and address social issues [6].

As the consequences of the pandemic unveiling, people’s subjective well-being is increasingly in the spotlight. There is growing evidence about the adverse effects of COVID-19, beyond contagion and economic contraction. One strand of the evidence shows a constant increase in the prevalence of conditions affecting the population’s mental health, such as depression, insomnia, burnout, and distress [7,8,9]. Likewise, reports increasingly uncover evidence for the strong prevalence of negative emotions such as worry, fear, and anxiety [10]. The lion’s share of the evidence of the consequences of the pandemic for people’s well-being is heavily concentrated in developed countries [11,12,13]. We know less about the consequences for wellbeing in developing countries facing larger budget constraints and social turmoil after the pandemic. In 2021, the Carnegie global protest tracker recorded over 25 significant protests related to the pandemic, many of them in Latin America, with violent protests in Argentina, Bolivia, Brazil, Chile, Colombia, Cuba, Haiti, Mexico, and Peru. The pandemic has brought to the forefront the wider income and social inequalities within and between countries, having significant implication for people’s well-being. 

In this paper, we seek to contribute to the research of the consequences of the pandemic for well-being by providing evidence from the global South. The is little information about the consequences of the pandemic in cities in Latin America, a region that experienced significant economic contraction, a large number of deaths, and an outburst of social turmoil [14]. To bridge this gap, we use information about life satisfaction and subjective well-being in Cali, the third-largest city in Colombia and the epicenter of the most significant outbursts of social unrest in recent history in Colombia. 

Our research is based on two hypotheses. First, that the pandemic may have adversely affected life satisfaction and subjective well-being of the population in Cali, the third largest city in Colombia, as has been shown to have happened in other contexts, particularly in developed countries. Addressing this issue contributes to the growing literature on happiness and subjective well-being in urban settings [15,16,17,18]. Second, we hypothesize that the adverse effects of the pandemic on life satisfaction may have varied across groups by sex and health status, as suggested by the previous literature on gender aspects of subjective wellbeing, including the context of the pandemic. Women report a higher increase in depression and worry and a reduction in their overall well-being during the pandemic [19,20]. At the same time, health is one of the more significant determinants of subjective well-being [21], and one of the primary direct effects of the pandemic. The information of this analysis aims at contributing to the discussion on how the knowledge of subjective well-being can inform better policymaking. We tackle subjective well-being information for better policymaking, given the scarce use of this type of information in Latin America [6]. 

To study the aftermath of the pandemic on subjective well-being and health, we use data for 2019 and 2020 to analyze changes during the pandemic. This analysis uses the city of Cali as a case study. The city deserves a particular focus, given its size (2.4 million inhabitants), the significant social and economic impact it experienced during the pandemic, and the fact that it was the epicenter of major social unrest in Colombia and Latin America after COVID-19. The data comes from two large surveys designed for measuring life satisfaction and subjective well-being in the city. In this analysis, we use a descriptive approach to create balanced samples by matching individuals in each group along an array of observable characteristics. This paper is organized as follows: Section 2, after this introduction, discusses the evidence available on life satisfaction and subjective well-being during the pandemic and the use of subjective well-being and life satisfaction metrics for policymaking. In Section 3, we present a description of the city and its context during the pandemic. Section 4 discusses the dataset, methods, and some caveats. Section 5 presents the analysis of our guiding research-questions, and Section 6 concludes with policy recommendations. 

## 2. Background

### 2.1. Life Satisfaction during the Pandemic

COVID-19, to a great extent, negatively affects all aspects related to our well-being. Multiple draconian measures implemented worldwide, such as lockdowns, social distancing, mobility restrictions, and closed borders, were a direct impediment to the social closeness that is pivotal for well-being and mental health [22,23]. The pandemic economic-impact included millions of jobs lost, economic contraction, and the economic pressures in countless households, all of which aggravated the consequences on people’s well-being [2]. The pandemic promoted an increased interest in the research of subjective well-being from several dimensions, ranging from economic stagnation [24,25,26], social determinants of health [27], deaths [28,29], variations in government response [30], trust in institutions [31], and benevolence and pro-social behavior [10]. The research available points to a large-scale negative impact on civil society and multidimensional spheres [32]. 

The long-lasting consequences of the pandemic on people’s well-being are yet to be seen. However, some predict that one of the pandemic’s most prolonged and severe consequences will be the deterioration of mental-health conditions [10,33]. The World Health Organization (WHO) in 2022 declared a massive 25% increase in depression and anxiety worldwide [34], calling for urgent measures in the health systems to prioritize mental health services and support. Alongside the WHO call, the research shows augmented stress, insomnia, and other conditions associated with poor mental health [9,35,36,37,38]. During the pandemic there were reports worldwide of a constant increase in poor mental-health conditions in diverse population groups across developed nations [10] with significant consequences in productivity and quality of life. Besides the prevalence of mental-health conditions, there is an increase in reports of people experiencing negative emotions. For instance, the World Happiness Report of 2021 informs of a 10% increase in people reporting being worried or sad the day before. The feeling of loneliness also increased during the pandemic, having a long-term negative impact on people´s physical and mental health [39]. 

Life evaluations, one of the overarching measures of subjective well-being, showed different trajectories. Some research reports significant reductions in life satisfaction [38,40], whereas other research reports a relatively constant life-satisfaction evaluation during 2020–2021, suggesting that the major impact of the pandemic is related to emotional changes –measured through negative affect—rather than the general evaluation people make about their lives [10]. The evidence suggests that life-satisfaction evaluations may have changed in line with the overall severity of the crisis, which differed greatly by country, depending on the infection peak and the general economic and social context.

Context also matters when evaluating the mental-health trajectories of the population. The evidence shows an increased number of web searches for terms associated with mental health deterioration in the U.S and Europe [41,42]. In Switzerland and France, it has been found that well-being measures (emotions and life evaluation) returned close to baseline a few months after lockdown [43]. 

A constant finding in the literature is the pivotal relevance of interpersonal trust and the ability to count on others for well-being, reinforcing the strong links between well-being and interpersonal relations [5]. Institutional trust also played a central role in the wellbeing of individuals during the pandemic. Evidence shows that mortality rates in European countries are related to trust in parliament, politicians and the legal system. In societies with high institutional trust there was a lower mortality rate [44] at the onset of the pandemic. High levels of institutional trust facilitate people´s acceptance of restrictive measures, government information or scientific advice [45]. Likewise, institutional trust has a strong positive correlation with wellbeing and life satisfaction, playing a mediating role in the relationship between government perception and individual subjective wellbeing and helping in difficult events [46]. For instance, in New Zealand, people´s psychological distress increased during the pandemic, while the trust in the government, national identity and sense of community improved, compared with before the pandemic [47]. 

The pandemic had an unequal effect across population groups, particularly when analyzing gender differences. Despite males having a higher probability of requiring intensive care and higher odds of death as a consequence of COVID-19 [48], reports show that women were more affected by a broad range of factors, including their professional careers [49], an increase in domestic violence [50] and increased health risks [51]. The evidence shows that women are particularly affected by increased mental-health disorders, and often experience anxiety, stress, and reduced job-satisfaction [12,19,20,52]. The pandemic also affected the well-being of those who perceived health-deterioration [40], but at different intensities when differentiating between those infected by COVID-19, the general population, and those in quarantine [53].

Although the consequences of the pandemic for subjective well-being are subject to ongoing research, the evidence shows that COVID-19 affects people´s life satisfaction and well-being, and is doing so in uneven ways for different groups of people and with significant differences, depending on context. During the pandemic, interpersonal trust and strong connections were substantial components of subjective well-being. The large bulk of the evidence provides information from developed countries, increasing the gap in the information available for understanding the long-term implications of the pandemic on well-being.

### 2.2. Subjective Well-Being and Life Satisfaction: Metrics for a Better Life

The metrics measuring subjective well-being are a relatively new addition to policy discourse. Subjective well-being gained relevance when Joseph Stiglitz, Amartya Sen, and Jean-Paul Fitoussi reported the limitations of measuring social advance based on economic metrics, particularly GDP growth [54]. One of the most salient messages from Stiglitz, Sen, and Fitoussi was “what we measure affects what we do”. Since then, multiple governments and organizations have discussed the relevance of using several dimensions of people’s lives that are key for well-being but not captured by economic metrics. In the last decade, subjective well-being has become a relatively common component of metrics in the developed world, defined as good mental-states and people’s evaluations of their lives [4]. 

The literature on subjective well-being is a growing field, showing solid links between well-being and physical and mental health [21,55,56,57,58,59], income [60], social relations and social capital [61,62,63], as well as government performance [64,65,66,67]. One important conclusion of this body of research is that non-monetary factors significantly impact people’s well-being. Good physical and mental health, close relationships, job satisfaction, and community involvement are far more important than economic measures, when it comes to people’s happiness [68]. Likewise, there is increasing evidence showing that subjective well-being strongly correlates with aspects related to the growing urbanization worldwide and the sustainability of cities. The built environment and urban-planning policies involving land use, transportation, urban design, and housing, directly impact people’s subjective well-being [69]. The accumulative evidence of the pathways, implications, and ramifications of the population’s well-being makes the study, its use, and subjective data, relevant for policy purposes. 

Since 2012, the Organization for Economic Co-operation and Development has promoted a wide range of metrics to be included by national statistical offices and population surveys to capture several dimensions of quality of life [4,5]. These metrics are intended to shed light on, among others, life satisfaction, affective states, physical and mental health, and interpersonal and government trust. Although many countries around the world are increasingly collecting data for measuring subjective well-being in the population, only a few are effectively using the data as a policy tool. According to the World Happiness Report 2022, only three countries (Bhutan, the United Kingdom and New Zealand) use well-being metrics in different stages of the policy cycle: monitoring, prioritizing, and policymaking [5]. 

The use of subjective well-being data to track quality-of-life changes and progress in cities became common in Latin America in the 1990s, after the creation of the Cómo Vamos –How are we Doing—system. The Cómo Vamos program is based on population surveys collected annually at city level. The program was pioneered by Bogotá, Colombia [69]. Following the lead of Bogotá, in recent decades many cities in Latin America are implementing programs and monitoring systems to evaluate the quality of urban-life in cities, relying extensively on subjective data. However, despite the improvement in data collection and information systems, it is unclear how the bulk of the data on subjective well-being collected in countries and cities across Latin America influences policymaking [6]. 

The quality of life in urban areas is gaining significant relevance for planning and resource allocation. Information about subjective well-being is also gaining a notorious significance in the context of rapid urbanization [70]. There is a clear link between the urban environment and the well-being of residents, and influencing how cities are planned, since it affects land use, transport systems, housing, and the general urban-design [70,71]. The use of objective and subjective data can be a powerful tool for evaluating urban public-goods, from security to green areas, and for informing policymakers on the better use of public resources [72]. Objective data cannot capture the many dimensions that affect the well-being of city habitants. Several relevant aspects of people’s lives are not captured with objective measures, such as a feeling of insecurity, relationships with neighbors, trust in public institutions, or the feasibility of transit within the urban environment. 

The pandemic provided an invaluable opportunity to open a policy window for a more extensive use of subjective well-being data in the policymaking context. The pandemic created the conditions to evaluate both what is most important for fostering a good life and what the role of governments is in providing conditions which enable the enhancement of the population’s well-being. As a consequence of the pandemic, people’s emotions [73], mental-health states [74], benevolence [5] and trust in institutions [31], gained increasing notoriety. The narrative change that caused the crisis generated by COVID-19 may provide a policy opportunity for a more extensive use of subjective well-being data in the policymaking domain, in developing countries that are less familiar with the use of this information. 

### 2.3. Cali and the Pandemic

Cali is the third-largest city in Colombia, with 2.4 million inhabitants [75]. Cali is one of the most complex cities a researcher can analyze, or where a policymaker can intervene. For the sake of illustration, and to deal with its complexities, let us check one single indicator: homicidal rates. During the 1990, homicidal rates in Cali were above 100 homicides per 100,000 habitants. In the past five years, until 2020, the indicator remained relatively stable, at approximately 50 homicides per 100,000 habitants [76]. These numbers need to be analyzed in context. Latin America is the most violent region globally, contributing to 30% of total homicides, with only 8% of the global population [77]. The average homicidal rate in a large city in the region is approximately 20 homicides per 100,000 inhabitants [78]. In recent years, Cali doubled the average, after having cut by approximately half, its homicidal rate of the 1990s. 

Before the pandemic, the city, like many other urban settlements in the region, was experiencing poverty reduction, middle-class growth, and improved access to public goods and services. Access to public services such as electricity had increased from 87% in 2006 to 91% in 2020 [79]. Life satisfaction was remarkably high in the city. Between 2014 and 2019, life satisfaction scores were 8.5 (on a scale of 0–10), not unlike national scores, but high compared with those in most cities in developed countries, where they range between 6 and 7.5 [2,80,81]. 

The pandemic significantly reversed the country’s positive trajectory in economic growth and basic-needs satisfaction. In 2020, income per capita fell by 8.6%, the incidence of monetary poverty increased from 35.7% to 42.5% and that of extreme poverty went from 9.6% to 15.1%. In the worst months of the lockdowns, total and formal employment fell to levels not seen since 2007 [82]. A wave of opinion surveys conducted by Ipsos between 26 March and 9 April of 2021 in 28 countries around the world showed that Peru, Colombia, and Chile were the countries where the largest shares of the population (89, 84 and 82%, respectively) considered that their countries were heading in the wrong direction [83]. With the ostensible objective of opposing a tax-reform bill introduced by the government, the largest labor unions in Colombia convened a general strike on 28 April 2021, which prompted the longest and most violent social turmoil the country had seen since 1948. For over six weeks, Cali became the epicenter of the riots, including violent confrontations with police forces and over 70 civilian deaths [84]. During most of the riots, there was a shortage of food, gas, and medical supplies, and groups of citizens were unable to leave their homes. The riots summoned an ample array of groups and sectors, including young students and the unemployed, who organized several points of “resistance”, taking control of large swathes of the city. These events took place against a backdrop of dissatisfaction with many aspects of people’s lives. Life satisfaction dropped from 8.5 in 2019 to 7.5 in 2020 [20], and mental health steadily worsened during 2020–2021, as reflected in opinion surveys. By July 2020, over 21% of the city’s residents declared their mental health was worse than the prior year; in November 2020, the share increased to 35%, and by January 2021, the proportion had reached 43% [85]. 

## 3. Data and Methods

This analysis aimed at testing two hypotheses:

1. The pandemic adversely affected life satisfaction and subjective well-being of the population in Cali;
2. The adverse effects of the pandemic on life satisfaction varied across groups, by sex and health status.

To investigate our hypotheses, we used data from surveys administered by the Observatory of Public Policies–POLIS- of Universidad Icesi. Since 2014, POLIS has carried out an annual population survey called CaliBRANDO, to measure subjective well-being in Cali. CaliBRANDO represents the demographic breakdown of the city’s race/ethnicity, gender, and socioeconomic composition, with a margin of error of 2.8% and a confidence level of 95% [86]. Each year the surveys collect over 1200 observations in face-to-face interviews conducted by trained pollsters. 

**Figure 1:**
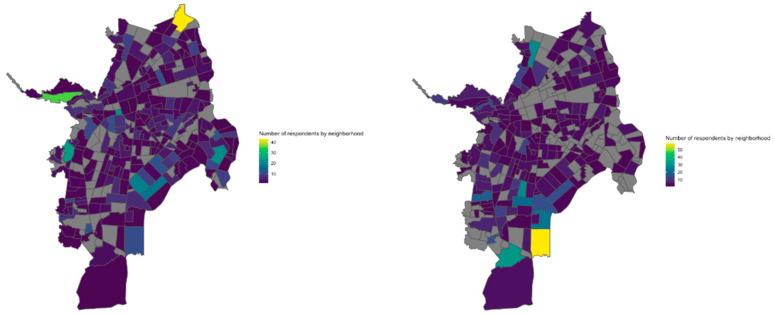
Survey respondents across the city for 2019 and 2020: the map on the left shows data from 2019, the map on the right shows data from 2020.

Due to the pandemic, the 2020 survey was not administered face-to-face, but online. The survey was gathered in partnership with a local newspaper [87], to reach a broader audience [88]. In total, 1000 Cali residents responded voluntarily to the survey (see Figure 1 for respondent´s distribution per year). Given the nature of online surveys, selection bias was a potentially serious problem. Respondents feeling a reduction in their well-being may self-select for the study, biasing the results. Likewise, it is important to highlight the fact that the sample from the 2020 survey was not representative of the population. To address these two important limitations, we used statistical methods that aimed to deal with the the fact that the sample-selection method does not assure that the observations are independently and identically distributed in a random fashion. Map 1 shows the respondent distribution for the surveys in 2019 and 2020, across the city.

Our aim was to assess how subjective well-being changed with the pandemic for our whole sample and by groups. We considered the following well-being dimensions: 

**Life satisfaction**: To measure life satisfaction, both surveys included a question taken from the OECD guidelines to measure subjective well-being in population surveys [4]. The question was “overall, how satisfied are you with your life as a whole these days”? The question scores ranged on a scale from 0–10, with zero meaning not at all satisfied, and ten completely satisfied. This question sought to capture the respondent´s evaluative judgment about his or her life.

**Affect balance variables**: These questions also came from the OECD guidelines, and were intended to characterize the affective state of the respondent the previous day [4]. Three questions were used in the survey: (i) “How happy did you feel yesterday?” (ii) “How worried did you feel yesterday?” and (iii) “How depressed did you feel yesterday?” The questions were scored on a 0–10 scale, with zero meaning not experiencing the feeling at all, and 10 experiencing the feeling all the time. 

**Mental health and social support**: As a proxy for mental health, we used the measures designed by the Centers for Disease Control and Prevention (CDC) for measuring “healthy days” [89]. We used the question “now thinking about your mental health, which includes stress, depression, and problems with emotions, for how many days during the past 30 days was your mental health not good?” For our analysis, we used a cut-off of three days. Those who declared having three or more poor physical and/or mental health days were coded as 1, otherwise they were coded as 0. In 2020, the survey included a question about whether the respondent (or a close relative) was infected with COVID-19. The surveys included the question “have you felt alone or without support lately?” with a yes-or-no response option.

**Personal satisfaction**: Respondents were asked about their satisfaction in six domains: family relations, job/employment, relationship with significant other, health, household economy, and income. These questions were scored on a 0–10 scale, with zero not satisfied, and 10 completely satisfied.

**Household economic perception**: This variable came from the question “compared to the last year, would you say that you are better, the same, or worse, economically speaking?” 

### Construction of Balanced Samples

To answer the research questions, we matched individuals with the same observable characteristics (that did not depend on the pandemic), using a matching-scores methodology [90]. The methodology consisted of finding a “twin” for as many individuals as possible in the 2020 survey, with individuals with similar observable characteristics in the 2019 survey. Thus, the propensity-score-matching approach was used to assess the impact of treatment [91]. The results of the propensity-score-matching comparison of all observations were obtained through a logit estimate called the propensity score. The average treatment effect on the treated (ATET) method was used with the smallest possible matching-distance obtained by the score (caliper = 0.01). However, two pairings were performed for the estimations focused across groups by sex and health status (see Tables 3 and 4). First, an estimation was carried out to match observations by year; second, the observations was matched according to the interest group (sex or health-status). Thus, this methodology used a similar difference-in-differences (DID) approach.

To that end, we used a broad array of socio-demographic variables (see Table 1): age group, socioeconomic strata, race/ethnicity, educational attainment and household composition. Some of these matching variables require some explanation. “Socioeconomic strata” refers to the household-stratification system used in Colombia, which is based on housing conditions and uses a scale from 1 to 6, the former being the most vulnerable [92]. However, given the distribution of the strata in the surveys for each year, the strata are aggregated into two categories: middle-low and low strata (SES 1, 2 and 3); middle-high and high strata (SES 4, 5 and 6). The “race/ethnicity” variable comes from a self-recognition question, which uses six categories: white, multi-racial, native, black, other, none. Using these variables, “minority” corresponds to black and native, “non-minority” corresponds to white and multi-racial, and “other” to other and none.

The construction of individually balanced samples ensured that group comparisons were valid, as they were not affected by compositional differences across groups (see Appendix A). Matching implicitly controlled for the difference in data collection (the 2019 sample was collected through face-to-face surveys, while the 2020 sample was an online survey). Since sampling was not random, this difference is a source of unbalance between the two samples.

## 4. Results

### 4.1. How Does the Pandemic Affect Life Satisfaction and Subjective Well-Being in Cali?

By 2019, life satisfaction in the city was high, at 8.5 on a 0–10 scale. Between 2014 and 2019, average scores of life satisfaction in the city did not change significantly. Compared to scores of approximately 7.5 reported for OECD countries [2], city residents were content with their lives. Cali´s numbers by 2019 mimicked national scores for life satisfaction [80,93] and the general rate of high life-satisfaction in Latin America [6]. 

Table 2 presents the landscape before and after the pandemic. Columns 1 and 2 present raw averages of the observations included in the matched samples of 2019 and 2020. Column 3 shows the mean difference between 2020 and 2019, computed using “tweens” (since some 2020 observations have more than one tween, the values of column 3 are not exactly the difference between columns 2 and 1). In 2019, the numbers of people feeling worried or depressed were relatively low, and in line with national figures [93,94,95]. On average, respondents declared that they had approximately 3.3 days of poor mental-health during the prior month, and 24% declared that they felt alone or without support. The lion’s share of respondents also declared that they were satisfied (over seven on the 0–10 scale) with different personal aspects, particularly family relationships and health. Only 10% felt that their household socioeconomic-condition was worse than the previous year. 

By 2020, the data shows a different story, regarding life satisfaction. All the variables analyzed record a significant reduction in subjective wellbeing with respect to the previous year. Life satisfaction dropped 1.6 units on the life-satisfaction scale (6.9 on average). This is a significantly large drop. Researchers have estimated a drop of one unit in life satisfaction as the equivalent of a 20% income-reduction [96]. As reported in many other countries, feelings of worry and depression increased during the pandemic [10]. In Cali, the largest increase was in feeling worried, with a spike of 2.4 units on a 0–10 scale. The average number of days feeling that mental health was lacking in the previous month was over eight days, increasing by four days, compared with 2019. Those reporting feeling alone and without support increased by 13 percentage-points from 2019 to 2020. Every aspect of personal satisfaction evaluated went down by approximately one unit. The proportion of respondents considering that the socioeconomic conditions of their household were worse than the previous year increased by 29%. 

The differences between 2020 and 2019 reported in column 3 show that all the well-being dimensions considered had changes significant at the 99.9 percent confidence-level.

### 4.2. Sub-Groups Analysis

#### 4.2.1. How Did Life Satisfaction Vary across Groups by Sex and Health Status?

Having shown that the pandemic brought very significant changes to the whole sample of individuals in all the well-being dimensions considered, we now want to see if those changes were more severe in some groups than others. In the tables that follow we focus on the difference-in-differences tests by gender and by health status. 

#### 4.2.2. Gender

We start by assessing gender difference-in-differences (DID), that is the differences between women and men in the difference between 2020 and 2019 in each of the well-being dimensions considered (Table 3). As in the previous table, the difference between columns 1 and 2 is not exactly column 3, as they do not include the same “twins” (column 1 comes from “twins” of women in 2020 and 2019; column 2 comes from “twins” of men in 2020 and 2019; column 3 comes from matching as many as possible of the 2020–2019 female “twins” with the 2020–2019 male “twins”). 

Life satisfaction had a substantial drop for both women and men, with no significant difference between both. Women, however, compared with men, did experience significantly less happiness and significantly more worry. With respect to mental health, compared with men, women experienced significantly fewer additional days of anxiety, depression, and stress, but the percentage of women who declared themselves as feeling alone or without support increased significantly more than that of men. In most of the dimensions of personal satisfaction, women and men experienced similar declines, but in their satisfaction with health, the decline was significantly steeper for women. However, in their satisfaction with income and their perception of the socioeconomic conditions of their households, women felt significantly more upbeat than men, although both gender groups felt worse than the previous year.

#### 4.2.3. Health Status

COVID-19 consequences are directly related to physical health. The symptoms of the infection negatively affect the physical health of those who are infected. The virus also underscores the importance of being physically fit and healthy, to reduce the severity of the infection. The pandemic, as never before, created the global anxiety of having the possibility of dying from a physical illness. The severity of the virus, and the direct effect on physical health, deserves special consideration when it comes to understanding the implications of the pandemic on people’s subjective well-being. 

To explore whether poor physical-health affected life satisfaction and well-being, we compared the differences between those who were ill with any condition (including COVID-19 in 2020), and those who did not report poor physical health (not ill). Table 4 presents the results; (as explained above, column 3 is not exactly the difference between columns 1 and 2, because the sets of observations matched are not the same).

A salient result was the difference in well-being by health status. Our results showed that those who were ill experienced a smaller reduction in life satisfaction and well-being. This is similar to findings from Singapore, where health satisfaction was unaffected through the pandemic [97]. Proxies for mental health (happy, worried, depressed, and the number of days of poor mental-health) showed a minor (but significant) difference, compared with those who were not ill in 2019 and 2020. The same pattern was observed with satisfaction in personal domains. We surmise two possible explanations. On the one hand, the pandemic may have provided individuals who fell ill with reasons for feeling grateful after a life-threatening episode, altering their perspective for judging their own situation. Alternatively, the pandemic may have altered the views of those who did not fall ill of the situation of others, by making them aware, for instance, of the difficulties experienced by those who fell ill or who, not having fallen ill, suffered economically or emotionally.

## 5. Discussion and Policy Recommendations

The pandemic was the major social, economic, and political shock experienced by current generations. The widespread economic insecurity, the disruption of every aspect of life, and the toll on the population’s mental health stagnated and pulled back social and economic gains for countries and individuals alike. An underrated consequence of the pandemic is the toll on happiness and subjective well-being. Most of the policy actions worldwide are heavily concentrated on economic recovery and strengthening the health system. But many other aspects of people’s lives should not be under the radar of policymakers, because of their devastating implications. 

A significant take from this evidence is the profound implication of the pandemic on mental health. Developing countries –including Colombia—spend negligible resources on mental-health diagnoses and treatment. According to OECD figures, developing countries invest approximately 2% of their healthcare costs in mental health [3]. Although some countries allocated a major share of resources to mental health as a consequence of the pandemic [33], in Colombia, at national and sub-national levels, there is no evidence of promoting mental-health programs or increasing budget allocation in this area. Another point from the evidence from Cali is the significant drop in people feeling alone or without support during the pandemic. This information is pivotal for governments, in order to center the policy agenda around increasing interpersonal and institutional trust. Latin America is one of the world regions with the lowest interpersonal and institutional trust, limiting the strength of the social fabric, economic growth, and the transparency of its institutions [97]. 

The use of subjective well-being data for policymaking in Latin America is an emerging process. To a great extent, policymakers do not know how to design, plan, and allocate public resources using the information provided from the analysis of subjective well-being research. The extensive evidence gathered worldwide during the pandemic shows the importance of good mental-health in understanding and promoting the population’s well-being. Women are more affected than men, which suggests that policies should promote better mental-health amongst the female population. Men are more affected by income, employment, and variables related to socioeconomic conditions. Given the pandemic impact on life satisfaction and well-being, the government’s response is falling short of compensating for the significant reductions. In the economic domain, policy intervention is focused on subsidizing firms and providing a basic income to the poor. Since total-income compensation is beyond the government’s means, and the generation of formal employment is a policy goal challenging to attain, attention to many other aspects of subjective well-being is necessary to promote the population’s well-being.

Some of the nuances and implications of the pandemic on people’s lives can be captured by the information provided with subjective well-being metrics. This information sheds light on the complexity of the policy interventions needed post pandemic, beyond buttressing health systems and pursuing economic recovery. Mental health, childcare, gender equality, social justice, and skills readaptation are some of the areas of policy action that have gained relevance because of their potential importance in buttressing the population’s well-being amid crises [3]. This global crisis may open an invaluable policy-window for promoting and discussing the broader use of subjective well-being data in the policymaking context.

## 6. Conclusions

In this analysis, we used data from the third-largest city in Colombia to assess how the pandemic affected people’s subjective well-being. Comparing the responses to a face-to-face representative survey implemented in 2019 (pre-pandemic) with those to an online survey carried out in 2020 (during the lockdown), we evaluated the consequences of the pandemic on people’s subjective well-being and mental and physical health. Since the online survey was possibly subject to sample selection-bias and was not representative of the population, we made use of econometric techniques to match the respondents with those of the face-to-face survey. 

This analysis has a two-fold purpose. On the one hand, we provide evidence about subjective well-being during the pandemic in one city in Latin America. To add to the ongoing research on subjective well-being during COVID-19, we give information on changes in well-being and how they affected two population-groups differently. On the other hand, we seek to contribute to the discussion of subjective well-being measures for better policymaking, post pandemic. 

Similar to findings in other contexts [38,40], our results show a significant reduction in life satisfaction during the pandemic. On average, Cali residents’ life satisfaction dropped by 1.6 units on a 0 to 10 scale, to a low level, given the historically high rates of life satisfaction in the country and the city in pre-pandemic times. Negative emotions and feelings increased for the entire sample. Worry, depression, and the number of days experiencing poor mental-health increased, as reported in other studies [5,10]. The proportion of respondents declaring feeling alone or without support increased during the pandemic, strongly correlating with life-satisfaction reduction. We also report significant decreases in satisfaction with personal domains, such as income, employment, and household economy.

The adverse effects of the pandemic were experienced differently by population sub-groups. As reported elsewhere [12,19,52], women experienced significant negative consequences in variables related to deteriorated mental-health. Worry, depression, and the number of days with poor mental-health were higher for women. While mental health was the primary consequence for women, men experienced a more considerable reduction in personal satisfaction related to income and employment. 

A major factor highlighted in our results is the difference in life satisfaction and subjective well-being between those who experienced and those who did not experience physical illness (including the COVID-19 infection) during the pandemic. Our analysis suggests that those who were ill during the pandemic had a smaller reduction in life satisfaction. We surmise that overcoming any illness, including COVID-19, which is life-threatening, may create a positive outlook and provide a different perspective on what is relevant in life. 

## Figures and Tables

**Table 1:** Descriptive statistics, 2019 and 2020.

Matching Variables	2019	2020
Gender		
Men	53%	56%
Female	47%	44%
Age		
18–30 years old	36%	64%
30+ years old	64%	36%
Socioeconomic Strata (SES)		
SES 1	26.3%	4.6%
SES 2	28.0%	11.1%
SES 3	30.4%	27.2%
SES 4	8.8%	22.6%
SES 5	5.9%	24.4%
SES 6	0.7%	10.0%
Ethnic		
Minority	34.2%	5.8%
Non-minority	62.3%	82.0%
Other	3.4%	12.2%
Education level		
High school	46.0%	6.0%
Professional	52.0%	86.2%
Postgraduate studies	2.0%	7.9%
None	0.0%	0.0%
Number of people in the household	3.6	3.5
Have children	66.7%	31.8%
Average income		
Less than 1 monthly minimum-salary (<230 USD)	21.0%	19.1%
Between 1 and 2 monthly minimum-salaries (231 USD–460 USD)	45.1%	26.0%
Between 2 and 4 monthly minimum-salaries (461 USD–920 USD)	9.3%	18.4%
Between 4 and 8 monthly minimum-salaries (921 USD–1840 USD)	3.5%	10.4%
More than 8 monthly minimum-salaries (>1840 USD)	1.0%	5.6%
No income	1.9%	1.6%
DK	18.2%	18.8%

**Table 2:** Life-satisfaction and subjective well-being measures in 2019 and 2020.

	Raw Mean		Mean Difference for Matched Observations
	2019 (1)	2020 (2)	2020 vs. 2019 (3)
Life satisfaction (0–10 scale)	8.5	6.9	−1.80
			***
**Affect balance**			
How happy you felt yesterday (0–10 scale)	8.3	6.9	−1.44
			***
How worried you felt yesterday (0–10 scale)	3.3	5.7	2.13
			***
How depressed you felt yesterday (0–10 scale)	1.8	3.6	2.06
			***
**Mental health and social support**			
Average number of days during the past month that mental health (anxious, depressed, stressed) was poor	3.3	8.5	4.84
			***
Percent of respondents declaring feeling alone or without support	24%	37%	0.13
			***
**Personal satisfaction**			
Satisfaction family (0–10 scale)	9.0	8.3	−0.87
			***
Satisfaction job/employment (0–10 scale)	7.8	5.9	−2.15
			***
Satisfaction relationship with significant other (0–10 scale)	7.7	6.7	−1.33
			***
Satisfaction health (0–10 scale)	8.3	7.7	−0.77
			***
Satisfaction household economy (0–10 scale)	7.6	6.6	−1.52
			***
Satisfaction income	7.1	5.5	−2.00
			***
**Household socioeconomic perception**			
Percentage of respondents considering the socioeconomic conditions in the household are worse than previous year	10%	39%	0.17
			***
**Sample size**	990	738	1728

*** *p* < 0.001.

**Table 3:** Gender differences on well-being in 2019 and 2020.

	2020 vs. 2019		Female vs. Male
	Female (1)	Male (2)	DID (3)
Life satisfaction (0–10 scale)	−1.76	−1.79	−0.08
	***	***	-
**Affect balance**			
How happy you felt yesterday (0–10 scale)	−1.49	−1.28	−0.28
	***	***	**
How worried you felt yesterday (0–10 scale)	2.32	1.88	0.47
	***	***	**
How depressed you felt yesterday (0–10 scale)	1.99	2.02	0.1
	***	***	-
**Mental health and social support**			
Average number of days during the past month that mental health (anxious, depressed, stressed) was poor	3.28	6.02	−2.28
	***	***	***
% Respondents declaring feeling alone or without support	0.17	0.09	0.11
	***	*	***
**Personal satisfaction**			
Satisfaction family (0–10 scale)	−0.81	−0.91	0.06
	***	***	-
Satisfaction job/employment (0–10 scale)	−1.97	−2.08	−0.05
	***	***	-
Satisfaction relationship with significant other (0–10 scale)	−1.29	−1.48	0.1
	***	***	-
Satisfaction health (0–10 scale)	−0.93	−0.56	−0.42
	***	***	***
Satisfaction household economy (0–10 scale)	−1.32	−1.69	0.13
	***	***	-
Satisfaction income	−1.75	−2.07	0.22
	***	***	-
**Household socioeconomic perception**			
% Respondents considering the socioeconomic conditions in the household are worse than previous year	0.17	0.15	0.11
	**	**	**
N Female	568	-	527
N Male	-	893	596

*** *p* < 0.001 ** *p* < 0.01 * *p* < 0.1.

**Table 4:** Differences in well-being from 2019 to 2020, by health status.

	2020 vs. 2019		Ill vs. Not Ill
	Ill (1)	Not Ill (2)	DID (3)
Life satisfaction (0–10 scale)	−1.39	−1.83	0.42
	***	***	***
**Affect balance**			
How happy you felt yesterday (0–10 scale)	−0.43	−1.62	0.88
	-	***	***
How worried you felt yesterday (0–10 scale)	1.52	2.05	0.21
	***	***	-
How depressed you felt yesterday (0–10 scale)	1.21	2.05	0.06
	*	***	-
**Mental health and social support**			
Average number of days during the past month that mental health (anxious, depressed, stressed) was poor	4.15	4.56	−0.2
	*	***	-
% Respondents declaring feeling alone or without support	0.10	0.11	0.002
	-	***	-
**Personal satisfaction**			
Satisfaction family (0–10 scale)	−0.77	−0.95	0.23
	-	***	*
Satisfaction job/employment (0–10 scale)	−2.06	2.19	−0.2
	***	***	-
Satisfaction relationship with significant other (0–10 scale)	−0.12	−1.48	0.77
	-	***	***
Satisfaction health (0–10 scale)	−0.72	−0.68	0.24
	*	***	*
Satisfaction household economy (0–10 scale)	−1.53	−1.47	−0.4
	***	***	**
Satisfaction income	−1.96	−1.94	−0.52
	***	***	***
**Household socioeconomic perception**			
% Respondents considering the socioeconomic conditions in the household are worse than previous year	0.54	0.07	0.3265
	***	-	***
N Ill	252	-	252
N Not Ill	-	1178	1074

*** *p* < 0.001 ** *p* < 0.01 * *p* < 0.1.

## Data Availability

Available data for this study can be accessed at Ref. [88].

## Appendix A

Diagnostic statistics are used for all comparisons to test the balance of covariates before and after matching (see Table A1, Table A2, Table A3, Table A4 and Table A5). First, a standardized difference of each covariate is differentiated by year. It is observed that when differentiating by year, each variable presents differences, which are corrected by performing the match. This correction means that the differences are approximately zero, showing no differences between the groups. Secondly, a variance ratio is performed for each covariate differentiated by year. The optimal result is that the variances are equal (value equal to 1), a result which is obtained after matching.

**Table A1:** Diagnostic statistics for Match in Table 2.

	Standardized Differences		Variance Ratio	
	Raw	Matched	Raw	Matched
Age				
30+ years old	0.58	0.00	1.01	1
Socioeconomic Strata (SES)				
Mid-high and High	0.96	0.00	1.88	1
Ethnic				
Minority	0.27	0.00	1.05	1
Education level				
Professional	0.79	0.00	0.48	1
Postgraduate studies	0.27	−0.00	3.66	1
Have children	−0.74	0.00	0.98	1

Note: The covariates are categorical variables, so the n − 1 groups of each are presented (the base category is omitted).

**Table A2:** Diagnostic statistics for first Match in Table 3 by Female.

	Standardized Differences		Variance Ratio	
	Raw	Matched	Raw	Matched
Age				
30+ years old	0.44	0.00	0.94	1
Socioeconomic Strata (SES)				
Mid-high and High	0.63	0.00	1.19	1
Ethnic				
Minority	0.10	0.00	0.99	1
Education level				
Postgraduate studies	0.17	0.00	1.84	1
Have children	−0.35	0.00	0.79	1

Note: The covariates are categorical variables, so the n − 1 groups of each are presented (the base category is omitted).

**Table A3:** Diagnostic statistics for first Match in Table 3 by Male.

	Standardized Differences		Variance Ratio	
	Raw	Matched	Raw	Matched
Age				
30+ years old	0.61	0.00	0.93	1
Socioeconomic Strata (SES)				
Mid-high and High	1.07	0.00	2.27	1
Ethnic				
Minority	0.29	0.00	1.08	1
Education level				
Professional	0.86	0.00	0.47	1
Postgraduate studies	0.25	0.00	3.84	1
Have children	−0.70	0.00	0.98	1

Note: The covariates are categorical variables, so the n − 1 groups of each are presented (the base category is omitted).

**Table A4:** Diagnostic statistics for first Match in Table 4 by Ill.

	Standardized		Variance Ratio	
	Raw	Matched	Raw	Matched
Age				
30+ years old	0.73	0.00	0.91	1
Socioeconomic Strata (SES)				
Mid-high and High	0.68	0.00	2.92	1
Ethnic				
Minority	−0.44	0.00	0.98	1
Education level				
Professional	0.72	0.00	0.29	1

Note: The covariates are categorical variables, so the n − 1 groups of each are presented (the base category is omitted). Since the sample for the sick is small, the covariate of having children is omitted, due to collinearity problems.

**Table A5:** Diagnostic statistics for first Match in Table 4 by not Ill.

	Standardized Differences		Variance Ratio	
	Raw	Matched	Raw	Matched
Age				
30+ years old	0.73	0.00	1.05	1
Socioeconomic Strata (SES)				
Mid-high and High	0.98	0.00	1.74	1
Ethnic				
Minority	0.24	0.00	1.01	1
Education level				
Professional	0.72	0.00	0.43	1
Postgraduate studies	0.26	0.00	3.0	1
Have children	−0.81	0.00	0.92	1

Note: The covariates are categorical variables, so the n − 1 groups of each are presented (the base category is omitted).
